# A Metabolic Activity Recovery of the Intestinal Microbiota in the Patients with Bronchial Asthma

**DOI:** 10.1155/2022/9902438

**Published:** 2022-09-19

**Authors:** Mariusz Ozimek, Vladimir Ivashkin, Oksana Zolnikova, Nino Potskherashvili, Konstantin Ivashkin, Natiya Dzhakhaya, Anastasia Kurbatova, Kira Kryuchkova, Victoria Zaborova

**Affiliations:** ^1^Institute of Sport, Department of Track and Field's Sports, University of Physical Education, 31-571 Krakow, Poland; ^2^Institute of Clinical Medicine, I.M. Sechenov First Moscow State Medical University (Sechenov University), Moscow, Russia; ^3^Vasilenko Clinic of Internal Disease Propedeutics, Gastroenterology, And Hepatology, I.M. Sechenov First Moscow State Medical University (Sechenov University), Moscow, Russia; ^4^Sports Adaptology Lab, Moscow Institute of Physics and Technology (National Research University), 141700 Institutskiy Pereulok 9, Dolgoprudniy, Moscow, Russia

## Abstract

**Background:**

It was established that the high biological diversity of intestinal microorganisms promotes the needed SCFAs production, which induces immune regulatory pathways and contributes to the anti-inflammatory response. *Study*. A group of 30 patients with allergic bronchial asthma (BA) were investigated in our study. All of the patients were tested for the presence of SIBO by the SCFA spectrum determination. For the SIBO treatment, 10 patients from the studied group were prescribed Rifaximinum with the 200 mg dose at 3 times a day for a week; the other 10 patients were prescribed Rifaximinum at the same dose, followed by the administration of the Lactobalance probiotic in capsules at 3 times a day for a month. A month probiotic course was assigned to the remaining 10 patients without SIBO, as part of the BA complex therapy. The SCFA studies were immediately carried out for all of the patients after the 1 month probiotic therapy course.

**Results:**

A normalization of the SCFA spectrum and anaerobic index for all of the studied patients were noted. Upon taking the probiotics, it was revealed in the patients without SIBO that the total content of fatty acids (*p* < 0.001), acetic and butyric acid (*p* < 0.001) had increased. The Rifaximinum course, followed by administration of the probiotics led to a decrease of the relative amount of isoacids and ratio of isoacids/acids in the studied patients as compared to the patients who had received Rifaximinum for the SIBO treatment only (*p* < 0.05).

**Conclusion:**

The obtained results demonstrate a potential opportunity of the drug influence on the active bacterial metabolites composition and amount in the intestinal biotope; as it was confirmed by the restoration of the intestinal microbiocenosis and microorganism habitat.

## 1. Introduction

The gastrointestinal tract contains a large, complex, and dynamic population of microorganisms which have noticeable influence on human health [1–4]. The relationship between the gut microbiota and its metabolic products with the host organism plays a key role in the immune system maturation, digestion, detoxification, production of vitamins, and prevention of the pathogenic bacteria adhesion, etc. [2, 5–7].

Short-chain fatty acids (SCFAs) have the greatest functional importance in the entire spectrum of bacterial metabolites (gases, hydroxy and dicarboxylic acids, biogenic amines, amino acids, etc.) [7–9]. In monocarboxylic acid, SCFAs can comprise of one to six carbon atoms. These acids (acetic, propionic, and oily) are formed from the saccharolytic microflora due to the bacterial fermentation of the indigestible fibers in the colon [7, 8].

The gut microbiota composition change or/and low biological diversity of microorganisms were associated with the SCFAs formation decrease. In turn, it has led to a polarization of the immune response towards type 2 T-helpers and formation of proinflammatory reactions, as it was shown in the experimental and clinical studies [5, 8–13].

The molecular mechanisms, which help SCFAs to reduce Th-2 mediated immune responses, are constantly being refined. A possibility of the SCFA influence on the activity of nuclear transcription factor, tumor necrosis factor-alpha and interaction with antigenic recognition receptors, G-protein receptors of polymorphonuclear neutrophils, etc. have also been established [3, 8, 9].

The SCFAs amount and ratio can reflect not only the composition of the intestinal biotope but also the functional activity of the bacterial representatives present [8, 9, 12, 13].

The aim of the research was to study the possibilities of correcting the short-chain fatty acids (SCFA) content and profiles in feces of the patients with allergic bronchial asthma.

## 2. Materials and Methods

### 2.1. Ethics Concern, Participants and Study Design

This prospective, randomized, controlled study was approved by the Local Ethics Committee of the Sechenov First Moscow State Medical University, Russian Federation (Protocol No. 05-18) on May 16, 2018 and was conducted in accordance with the Declaration of Helsinki. The aim of the study was explained to the potential participants and the signed consents were obtained from the patients before their enrolment in the study.

**Figure 1:**
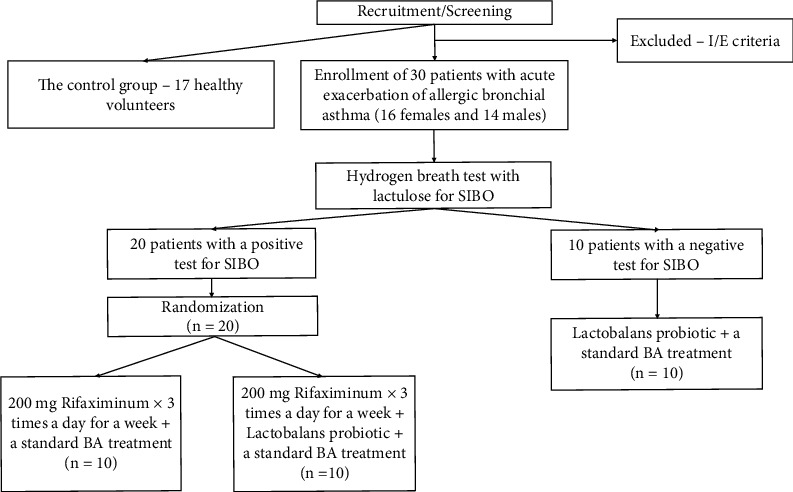
The flow chart of the study design (description in the text).

Between June 2018 and October 2018, we enrolled 30 patients with acute exacerbation of allergic bronchial asthma (16 females and 14 males) with the average age of 37.7 ± 10.1 years and anamnesis duration of 11.4 ± 8.7 years (Figure 1). All patients have been treated at Vasilenko Clinic of Internal Disease Propedeutics. Inclusion criteria were persistent-moderate severity of asthma with the consumption of combined preparations containing the long-acting beta-2-adrenergic agonists and inhaled glucocorticoids (a standard basic therapy for a treatment of bronchial asthma). Exclusion criteria were taking antibacterial drugs over the previous 3 months, as well as any probiotics and prebiotics, the proton pump inhibitors and hypoglycemic drugs in the study. All of the patients underwent the generally accepted clinical and laboratory tests, including blood, sputum, and urine tests and biochemical blood tests. The level of immunoglobulins (class A, G, and E), C-reactive protein, X-ray examination of the lungs, and respiratory function had been defined for the patients before the investigation.

All of the patients were additionally tested using a hydrogen breath test with lactulose for the presence of the syndrome of intensive bacterial overgrowth (SIBO) in the small intestine. According to the obtained results of the test, 20 patients with a positive test for SIBO were randomly divided into 2 groups (10 people each group). The randomization process was performed using a computer-generated randomization table. The remaining 10 patients with a negative test for SIBO were assigned to the third group.

The first group was consisted of the 10 patients with a positive test for SIBO (SIBO (+)) and was prescribed Rifaximinum of 200 mg dose, 3 times a day for a week together with a standard BA treatment. The second group was consisted of the 10 patients with a positive test for SIBO (SIBO (+)) also, but they took Rifaximinum of 200 mg dose, 3 times a day for a week together with the prescription of Lactobalance probiotic. The Lactobalance probiotic was prescribed in capsules, to be taken 3 times a day for a month, where each capsule had contained three billions of probiotic microorganisms (3.0 × 10^9^ CFU/capsule at least): there were *Lactobacillus gassery KS-13, Lactobacillus gasser LAC-343, Lactobacillus ramnosus LCS-742, Bifidobacterium bifidum G9-1, Bifidobacterium longum MM-236, Bifidobacterium longum MM-236 M, Bifidobacterium infantis M-63, Bifidobacterium breve M16V type T,* and *Bifidobacterium lactis B1-04* in the capsule. The third group was consisted of the 10 patients with allergic asthma without detected SIBO (SIBO (-)) and they took the Lactobalance probiotic, as it was part of a standard therapy for the BA treatment in the group. The control group included 17 healthy volunteers (the average age of 37.6 ± 9.5 years, with 9 females and 8 males). All of the studied groups were comparable among themselves by age (*p* > 0.05) and gender (*p* > 0.05). The patient groups were comparable in terms of anamnesis (*p* > 0.05) and disease severity (*p* > 0.05).

The study of SCFA was performed for a month after the beginning of the treatment (i.e., at the end of the probiotic administration).

### 2.2. Diagnosis

To diagnose the syndrome of intensive bacterial overgrowth (SIBO) in the small intestine, a hydrogen breath test with lactulose Gastrolyzer (Bedfont Scientific Ltd) was performed.

The SCFA spectrum was determined by gas-liquid chromatographic analysis. The study was carried out on the “Chromos GH-1000” gas chromatograph with a flame ionization detector. The absolute and relative contents of acetic (C_2_), propionic (C_3_), and butyric (C_4_) acids, the level of isoacids, and the ratio of isoacids to acids (isoCn/Cn) as well as the values of the anaerobic index (AI = (C_3_ + C_4_)/C_2_) were evaluated [14].

### 2.3. Statistical Analysis

Statistical analysis of the results was carried out using the program Statistica 10 (StatSoft Inc., USA).

## 3. Results of the Investigation

At the first stage of the investigation, we have studied the level and spectrum of SCFA in feces of the patients with allergic and nonallergic BA and a group of healthy volunteers. The results of that study were published in the Journal Clinics and Practice 2019, No. 9. A significant decrease in the total content of SCFA (*p* < 0.001) as well as the absolute concentrations of acetic, propionic, and butyric acids (*p* < 0.001) in patients with BA (regardless of the disease phenotype) were found. The altered spectrum of SCFA was revealed: anaerobic metabolism of microflora was determined for 84% of the patients, and aerobic metabolism of microflora was determined for 16% of the studied patients. The values of the anaerobic index varied in accordance with the metabolic profile of SCFA. A sharp shift of the index towards negative values for the anaerobic type (*p* < 0.01) and to the zone of opposite values for the aerobic type [10] was recorded.

Having obtained the first stage results of our study, we have attempted to correct the composition of the intestinal microbiota in the group of the patients who suffered from allergic bronchial asthma.

According to the study results, it was found that the prescription of the therapy for the SIBO treatment contributed to a decreasing of the isoacids amount, and the level of propionic acid in the patients had been taking a prolonged therapy (antibiotic+ probiotic). The level of the remaining SCFAs as well as the total content of ones did not change after the treatment in these groups (Table 1).

Patients of the third group (SIBO (-)), which were not needed to prescribe an antibacterial drug, a significant increase of the total content was revealed for SCFAs, acetic, and butyric acids. A decrease of the isoacids amount was also noted (Table 2).

**Figure 2:**
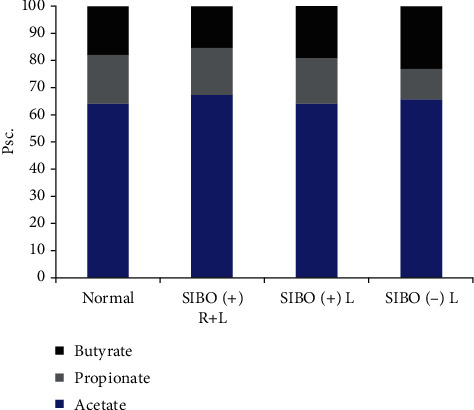
The short chain fatty acids profile in the studied groups.

We had been determining the spectrum of SCFA during the study. After the aimed therapy for the microbiota correcting of the intestinal biotope, the ratio of SCFA to acetic : propionic : butyric was indistinguishable in all of the studied patients from the control group (Table 3, Figure 2). Those results were obtained for the control group at the first stage of our study (it was published in Clinics and Practice 2019, No. 9).

**Figure 3:**
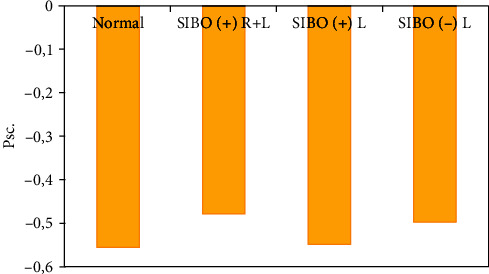
The anaerobic index values after the patients treatment.

In the group of the patients who had taken Rifaximinum + Lactobalance, the relative content of isoacids and ratio of isoacids/acids significantly decreased as compared with the control group (*p* < 0.001) and group of the patients who had taken Rifaximinum only (*p* < 0.05). The decrease of these indicators was also detected for the patients who had been taking Lactobalance only, as it was also confirmed by the preepithelial and epithelial levels improvement of intestine in a response to the probiotic therapy. The treatment used also contributed to a normalization of the anaerobic index values in all the studied groups (*p* > 0.05) (Figure 3).

## 4. Discussion

A commensal microbiota constantly interacts with the immune system training immune cells to respond to antigens [1, 2, 7]. Macrophages, neutrophils, and dendritic cells as well as other types of cells including epithelial cells which are in close contact with the microbiota, thus activating the membranes and intracellular proteins which recognize the antigens [3, 7, 9].

It was established that the high biological diversity of intestinal microorganisms promotes the needed SCFAs production, which induces immune regulatory pathways and contributes to the anti-inflammatory response [6–9]. On the contrary, the low biological diversity of intestinal microorganisms is associated with a decreasing of the SCFAs production, which leads to the immune response polarization and its shift towards the type 2 T-helpers [1, 4, 6–9, 12].

As mentioned earlier, the SCFA's change indicates on the microbiocenosis violations of the intestinal biotope in the patients with BA [8, 9, 11]. It may be one of the reasons for the disease development and perhaps, because of the changes in the CO2 and H2 excretion as a result of disturbances in ventilation and perfusion of the lungs, leads to a shift in the redox potential of the intraluminal intestinal environment in the patients with BA disease [11]. The SIBO presence in the small intestine in most of the studied patients with allergic BA (according to our data - 63%) indicates more pronounced disorders of the microbial community and can be considered as a factor aggravating the BA course [10, 11].

The result of the presented study is the revealed changes in the synthesis of SCFAs during the BA treatment with SIBO in the two schemes (antibiotic and antibiotic + probiotic), as well as against the background of the probiotic preparation prescription for the patients without SIBO.

The consumption of probiotic Lactobalance after a course of Rifaximinum led to a decrease in the relative amount of isoacids and the ratio of isoacids/acids in comparison with patients who received Rifaximinum only for treatment of SIBO (*p* < 0.05). Similar changes (*p* < 0.001) were also obtained in the patients who had taken a probiotic preparation as part of the BA complex therapy. The obtained results reflect the positive effect of probiotic cultures on the state of the intestinal mucosa and indicate the restoration of the preepithelial and epithelial layers in general, and the composition of the microbiota with proteolytic activity has been normalized also [4, 5, 8, 9].

No changes were detected in the content of SCFA in feces after a month of the SIBO treatment. In our opinion, it is due precisely to the presence of SIBO in the small intestine, which reflects a significant destabilization in the microbial community of the intestinal biotope. The antibacterial drug use for these patients, on the one hand, led to selective contamination of the small intestine, which ultimately contributed to the SCFA profile normalization. On the other hand, it was not possible to increase the SCFA formation in bacteria at the indicated time interval.

In patients of the third group, who received the probiotic as a part of the complex BA therapy, a significant increase of the SCFA total content (*p* < 0.001), acetic, and butyric acid (*p* < 0.001) was revealed for a short observation period, which demonstrated the positive effect of the probiotic Lactobalance preparation on the activation of endogenous flora and normalization of its metabolic activity.

In all patients, after the treatment had been aimed to modify the intestinal microbiota composition, the SCFA spectrum and values of the anaerobic index were similar to the consequent values for the control group (*p* > 0.05).

Thus, our results demonstrate the potential possibility for the drug exposure on the composition and active bacterial metabolites amount in the intestinal biotope. The presented results indicate that further study of the intestinal microbiota changes will be needed with regards to BA disease. Obviously, to achieve the reference values of the SCFA amount, a more durable period of the probiotic therapy will be needed for that. So far, we cannot answer the following questions: would the SCFA amount and spectrum change if the patients with BA and SIBО have been prescribed a probiotic drug only? Does the microflora correction influence the asthma course? It will be evaluated in a result from the further monitoring of these patients.

## Figures and Tables

**Table 1:** The level of SCFAs in patients with allergic bronchial asthma before and after the SIBO treatment.

Parameter	Allergic asthma SIBO (+), before the treatment	Allergic asthma SIBO (+), after the treatment	
		Rifaximinum+Lactobalance	Rifaximinum
Total content, (mg/g)	3.28 [1.14; 5.43]	2.59 [2.05; 3.13]	2.07 [1.95; 7.05]
Acetic acid (C_2_), (mg/g)	1.66 [0.54; 2.78]	1.58 [1.23; 1.92]	1.25 [0.04; 4.5]
Propionic acid (C_3_), (mg/g)	0.76 [0.26; 1.25]	0.19^∗∗^ [0.15; 0.23]	0.24 [0.02; 0.73]
Butyric acid (C_4_), (mg/g)	0.54 [0.20; 0.87]	0.56 [0.42; 0.69]	0.42 [0.01; 1.6]
Isoacids (Сn), (mg/g)	0.23 [0.1; 0.37]	0.12^∗^ [0.08; 0.15]	0.11^∗^ [0.08; 0.12]

Mann–Whitney test ^∗^*р* < 0.05, ^∗∗^ *р* < 0.01 in compare with the group before the treatment.

**Table 2:** The level of SCFAs in patients with allergic bronchial asthma before and after treatment with the probiotic.

Parameter	Allergic asthma SIBO (-), before the treatment	Allergic asthma SIBO (-), after the treatment	*р*
Total content, (mg/g)	1.84 [1.48; 2.21]	4.42 [3.8; 5.01]	< 0.001
Acetic acid (C_2_), (mg/g)	0.95 [0.62; 1.29]	2.88 [1.15; 4.62]	< 0.001
Propionic acid (C_3_), (mg/g)	0.36 [0.19; 0.52]	0.45 [0.02; 0.88]	< 0.001
Butyric acid (C_4_), (mg/g)	0.23 [0.11; 0.35]	1.01 [0.06; 1.96]	< 0.001
Isoacids (Сn), (mg/g)	0.39 [0.07; 0.71]	0,03 [0.0; 0.05]	< 0.05

**Table 3:** The SCAFA's spectrum after the treatment in the studied groups.

Acids	Control group	АА SIBO (+)		АА SIBO (-), Lactobalance
		Rifaximinum + Lactobalance	Rifaximinum	
Acetic acid (C_2_), pcs.	0.64 [0.55; 0.73]	0.67 [0.66; 0.68]	0.64 [0.56; 0.71]	0.66 [0.59; 0.73]
Propionic acid (C_3_), pcs.	0.19 [0.17; 0.2]	0.18 [0.17; 0.19]	0.17 [0.12; 0.19]	0.11 [0.08; 0.12]
Butyric acid (C_4_), pcs.	0.17 [0.14; 0.21]	0.14 [0.12; 0.15]	0.19 [0.11; 0.28]	0.23 [0.18; 0.27]
Isoacids (Сn), pcs.	0.059 [0.05; 0.06]	0.04^∗∗^ [0.03; 0.05]	0.09^ [0.06; 0.26]	0.008^∗∗∗^ [0.002; 0.001]
IsoCn/Cn, pcs.	0.45 [0.35; 0.54]	0.17^∗∗∗^ [0.12; 0.23]	0.50^[0.4; 1.4]	0.035^∗∗∗^ [0.01; 0.06]
Anaerobic index, pcs.	-0.55 [-0.64; -0.46]	-0.48 [-0.49; -0.46]	-0.55 [-0.74; -0.36]	-0.50 [-0.65; -0.34]

Mann–Whitney test ^∗∗∗^*р* < 0.001, ^∗∗^ *р* < 0.01 in compare with the control group; *^p* < 0.05*in compare with* Rifaximinum + Lactobalance.

## Data Availability

The data used to support the findings of this study are presented in the dissertation, which you can find at the link https://www.dissercat.com/content/mikrobiota-kishechnika-i-dykhatelnykh-putei-kak-patogeneticheskoe-zveno-bronkhialnoi-astmy.
